# The Grape Gene Reference Catalogue as a Standard Resource for Gene Selection and Genetic Improvement

**DOI:** 10.3389/fpls.2021.803977

**Published:** 2022-01-17

**Authors:** David Navarro-Payá, Antonio Santiago, Luis Orduña, Chen Zhang, Alessandra Amato, Erica D’Inca, Chiara Fattorini, Mario Pezzotti, Giovanni Battista Tornielli, Sara Zenoni, Camille Rustenholz, José Tomás Matus

**Affiliations:** ^1^Institute for Integrative Systems Biology (I2SysBio), Universitat de València-CSIC, Valencia, Spain; ^2^Department of Biotechnology, University of Verona, Verona, Italy; ^3^INRAE, SVQV UMR-A 1131, Université de Strasbourg, Colmar, France

**Keywords:** grapevine, database, gene characterisation, gene repository, integrape

## Abstract

Effective crop improvement, whether through selective breeding or biotech strategies, is largely dependent on the cumulative knowledge of a species’ pangenome and its containing genes. Acquiring this knowledge is specially challenging in grapevine, one of the oldest fruit crops grown worldwide, which is known to have more than 30,000 genes. Well-established research communities studying model organisms have created and maintained, through public and private funds, a diverse range of online tools and databases serving as repositories of genomes and gene function data. The lack of such resources for the non-model, but economically important, *Vitis vinifera* species has driven the need for a standardised collection of genes within the grapevine community. In an effort led by the Integrape COST Action CA17111, we have recently developed the first grape gene reference catalogue, where genes are ascribed to functional data, including their accession identifiers from different genome-annotation versions (https://integrape.eu/resources/genes-genomes/). We present and discuss this gene repository together with a validation-level scheme based on varied supporting evidence found in current literature. The catalogue structure and online submission form provided permits community curation. Finally, we present the Gene Cards tool, developed within the Vitis Visualization (VitViz) platform, to visualize the data collected in the catalogue and link gene function with tissue-specific expression derived from public transcriptomic data. This perspective article aims to present these resources to the community as well as highlight their potential use, in particular for plant-breeding applications.

## From Functional Genomics to Genetic Improvement

Online tools and databases are key to harness the potential offered by genomic advances to both research and industry. The Arabidopsis Information Resource (TAIR) for the *A. thaliana* community represents the most successful example in the plant field (Berardini et al., 2015), providing up-to-date biological information on individual genes as well as related resources such as single-gene mutant collections. Other examples of equivalent resources are the Sol Genomics Network (SGN) for Solanacea family members such as tomato, potato, and tobacco (Fernandez-Pozo et al., 2015), the Rice Annotation Project Database (RAP-DB) (Sakai et al., 2013), and the Maize Genetics and Genomics Database (maizeGDB) (Woodhouse et al., 2021). Although some resources are available for grapevine, there is plenty of room for improvement.

The first release of the highly homozygous *Vitis vinifera* cv. PN40024 reference genome assembly (Jaillon et al., 2007) and its subsequent annotation updates, including 12X.CRIBI-V1 and 12X.VCost.v3 (Canaguier et al., 2017), represent hallmarks in the grapevine field as they paved the way for gene characterisation studies at the genome level. Moreover, there have been key advances in assemblies of contiguous and phased diploid genomes of grapevine cultivars of greater agricultural importance such as cv. “Cabernet Sauvignon” (Chin et al., 2016; Minio et al., 2019b), cv. “Carménère” (Minio et al., 2019a), and even clones of cv. “Zinfandel” (Vondras et al., 2019). The increasingly available data allows for a pangenomic understanding of the grapevine genome, which in time will be the ultimate goal.

**FIGURE 1:**
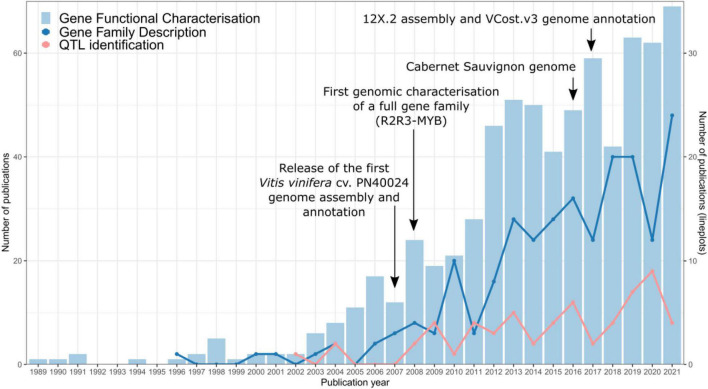
Publication trends of gene and gene family characterisation studies in grapevine. The PN40024 genome assembly and annotation releases (Jaillon et al., 2007; Canaguier et al., 2017), the first genome-assisted gene family study (Matus et al., 2008), and the phased diploid Cabernet Sauvignon genome assembly (Chin et al., 2016) are included as milestones in grape genomics. Data were collected from NCBI using three different queries for each category. Gene functional characterisation query: (*vitis vinifera[Title/Abstract] OR grapevine[Title/Abstract]) AND (gene [Title/Abstract]) AND (function[Title/Abstract] OR role[Title/Abstract] OR characterization[Title/Abstract] OR characterisation[Title/Abstract])*. Gene family description query: (*vitis vinifera[Title/Abstract] OR grapevine[Title/Abstract]) AND family[Title/Abstract] AND phylogenetic [Title/Abstract]*. QTL identification query: (*vitis vinifera[Title/Abstract] OR grapevine[Title/Abstract]) AND QTL [Title/Abstract] AND (gene [Title/Abstract] OR function [Title/Abstract] OR map [Title/Abstract])*.

In recent years, an increasingly large number of scientific articles related to gene functional characterisation and gene family description have been published in this species (Figure 1), most often using the reference PN40024 gene IDs. This wealth of information promises to be instrumental in crop improvement strategies. For instance, secondary metabolism genes have roles in grapevine defence and responses to abiotic and biotic stresses, but also act as quality parameters since they affect fruit colour and aroma attributes which then have an impact on produced wines. Therefore, these genes are of great interest in plant breeding programs. A growing population and the forecasted effects of climate change highlight the importance of crop genomics and plant research in contributing to the long-term goal of increasing sustainability in agriculture whilst not compromising food supply. A compendium of gene identities and their currently described functions is hence a basic start point for gathering functional genomics research carried out by plant research communities. In the case of grapevine, this will allow a well-informed selection of genes in a wide-range of applications.

## Development of a First Grape Reference Gene Catalogue

With the aim of supporting the grapevine and a wider fruit crop community, we have developed the grape reference gene catalogue. Devised as an updateable resource it is currently on its 2.3 version and it holds more than 2,000 genes referring to the 12X.1 and 12X.2 cv. ‘‘PN40024’’ genome assemblies. The basic structure of the catalogue consists on a single unique VCost.v3 gene ID per row, representing the latest model for a particular gene, matched by a unique gene symbol in a different column. The latest gene catalogue is found at the Integrape COST Action website^1^. Moreover, an updated VCost.v3 gff3 file, in which the catalog’s gene symbol information has been integrated, is also provided. Given the ongoing gene assembly and annotation improvements on behalf of the grapevine community, an effort has been made in providing comprehensive and up-to-date VCost.v3 to CRIBI V1 IDs correspondences, which are completely different in their code and naming structure. Where multiple V1s correspond to the same VCost.v3 gene ID, these have been provided in the same field separated by semicolons. This effort is particularly important to maintain a connection to previously published literature working with previous annotation versions. This basic structure will be updated after the release of the 40X.v4 assembly (Velt et al., in preparation), by providing unique V4 gene IDs per row and their matching gene symbols. In the meantime, where there is recent evidence of a VCost.v3 gene model splitting into two V4 genes, a composite gene symbol is provided [e.g., Laccase (LAC) 9/10]. Moreover, in cases where two VCost.v3 gene models merge into a single V4 gene, a suffix is provided to show that these V3 gene models are actually corresponding to a single gene [e.g., LAC40(i) and LAC40(ii)].

Besides gene symbols and V3/V1 IDs, the catalogue also provides gene name/symbol synonyms, full descriptive gene names, and the pathway or gene family to which a gene belongs as well as their respective group or subgroup in case of inner classification. The fact that metabolic reactions are typically sequential and normally catalysed by specific enzymes has allowed the organisation of the catalogue’s enzyme-coding genes according to their position within metabolism, which allows a hierarchical view of pathways and their underlying reactions. A further thing to note is that there are usually several isoenzymes for each metabolic reaction and these are always grouped together. For instance, following this type of ordering, shikimate pathway enzymes (primary metabolism) appear first in the catalogue according to reaction order. They are subsequently followed by early phenylpropanoid pathway steps, which then branch into lignin, stilbenoid and flavonoid synthesis. This sort of pathway-based ordering was not extended to other types of genes such as transcription factors as they often participate in several pathways. Hence, transcription factors are just organised in terms of their gene families. To further categorise genes, the group and subgroup columns are included, which can be redundant for gene families where subgroups do not exist or have yet to be defined. So far there are 581 enzymes classified into pathways, 204 unclassified enzymes and 880 transcription factors amongst other gene types. Since the regulation of metabolism is known to occur mainly at the transcriptional level, a good knowledge on the existing transcription factor gene families is bound to be helpful in plant breeding strategies.

A further aim of the catalogue is to indicate a confidence level for the stated gene function based on the currently available literature evidence. A 6-tier validation level system was developed to reflect different types of supporting evidence. The lowest level is for hypothetical functions simply based on similarity to other proteins (e.g., through BLAST or equivalent programs). The next level represents putative functions where a family-level gene characterisation may be complemented with some sort of expression data. Level three is granted where there is further statistical evidence in the form of gene co-expression networks or correlation to metabolite profiles. QTL mapping results correspond to validation level four. Level five corresponds to stable or transient transgenesis experiments in other plant species. The highest validation level, six, corresponds to the same type of experimental validation as level five but in *Vitis vinifera*. Biochemically validated enzymes in other species such as *Escherichia coli* or *Nicotiana benthamiana* are also given the maximum level of validation, as same-species validation is considered impractical and unnecessary. Finally, the literature supporting such validation levels is cited in separate columns depending on whether the publication experimentally demonstrates a functional association or whether it describes a gene family through *in silico* methods. References and DOIs are provided for each publication and in the case of gene family characterisations the phylogenetic method used must be indicated.

A single genome assembly does not faithfully represent the gene function wealth present in a species such as grapevine. The highly homozygous state of PN40024 (leading to potential gene loss) and the structural variation between cultivars and the genetic importance of those differences at the phenotypic level means that genomic approaches are destined to a pangenome approach. Currently, we have made an effort to provide gene catalogue gene correspondences to the phased diploid genome assembly of cv. Cabernet Sauvignon (Minio et al., 2017). Conversions between PN40024 and CS08 Cabernet Sauvignon gene IDs were obtained from a conditional reciprocal best BLAST using the CRB-BLAST software with default parameters (Aubry et al., 2014). This can be seen as the first step toward a future pangenome version of the catalogue, which would connect published gene function data to new and updated genome assemblies from different cultivars, as well as the current PN40024 reference.

There is a noticeable gap between the ≈2,000 genes in the catalogue and the 41,733 protein coding genes in *Vitis vinifera* according to VCost.v3 (Canaguier et al., 2017). However, this gap is suspected to be somewhat smaller according to recent protein-coding gene estimates for the new PN40024 genome assembly (40X.v4), which are around 35,000 genes suggesting that the VCost.v3 genome annotation overestimated the real number. Nevertheless, the sizeable gap will get progressively smaller as grapevine research advances and more gene families are being characterised on a monthly basis. All of these families will be incorporated in future versions of the catalogue. Although the initial effort attempted to cover already published literature, a lot of published literature remains to be examined in order to enrich the gene catalogue. This may be especially true of publications dealing with genome annotation accessions prior to CRIBI V1. Since the catalogue will likely be in constant development, we encourage the grapevine community to participate. To this end, we have provided an online system through which any researcher can add gene members to the catalogue by filling and submitting the provided form (see text footnote 1). The minimum information requested would be V3 IDs, gene symbols, validation levels and the relevant citation information.

## Gene Cards

Gene Cards is an app developed within the Vitis Visualization (VitViz) platform^2^ : a suite of bioinformatic tools working on up-to-date *Vitis vinifera* datasets Gene Cards integrates catalogue information with RNA-Seq transcriptomic datasets providing a biological summary sheet on an individual gene basis. Individual genes can be found through the provided search bar in the form either through their gene symbol, V3 or V1 IDs. The resulting summary includes information from the catalogue and other sources as well as providing an interactive tissue-specific view of gene expression. Information is provided for every annotated gene in the genome, however, a greater level of detail is associated to catalogue genes.

The transcriptomic datasets shown in Gene Cards have been derived from public SRA data with the objective of being as comprehensive as possible. A general query (“vitis vinifera”[Organism] AND ILLUMINA[Platform]) NOT (Bisulfite-Seq[Strategy] OR GENOMIC[Source] OR METAGENOMIC[Source] OR DNase-Hypersensitivity[Strategy] OR Bisulfite-Seq[Strategy] OR WGS*[Strategy] OR ncRNA-Seq[Strategy] OR WCS[Strategy] OR degradome OR miRNA-Seq[Strategy] OR small RNA[Title] OR sRNA[Title]) was used as a first filter to obtain 4,815 runs corresponding to mRNA transcriptome sequencing experiments. The first search was carried out on the 28th of April 2021 and the number of SRA runs are expected to grow as periodic updates take place. This will be especially important for tissue categories for which there are not as many SRA runs yet. The metadata of these runs was used to perform both automatic and manual sorting into nine different tissue categories; leaf, fruit, flower, bud, tendril, stem, seed, root, and wood. Further filters, applied to the datasets, included removing runs corresponding to mixed tissues, making sure sRNA/ncRNA datasets were removed and setting a minimum of ten million successful alignments per run and four runs per BioProject. A total of 2,737 SRA runs were included in the Gene Cards application. The data are interactively represented in the form of tissue-specific box-plots in which each dot corresponds to a single SRA run, which upon selection displays both the SRA run and BioProject IDs. The expression values are shown in the form of log (FPKM + 1) and the fact that particular points can be traced to their original experimental conditions permits the exploration of many biological aspects, which will often be relevant in plant breeding strategies. This app is designed to assist the wider grapevine community, whether involved in basic research, biotechnology or agricultural applications.

## Discussion

Previous gene catalogue attempts can be found in the literature, such as the summary of 39 functional genes related to wine aroma (Lin et al., 2019). An earlier version of the grape reference catalogue has already been of use in Orduña et al. (2021). The cistromes of three R2R3 Mybs were examined through DNA affinity purification (DAP-Seq) and the subsequent biological interpretation was facilitated by the gathered gene function information. This highlights the potential benefit of such a resource regarding genome-wide biological questions. With its over 2,000 genes, we expect to cover key gene families with respect to genetic improvement strategies in grapevine, since genes related to different plant stresses were specifically sought for. In fact, genomic analysis in *Vitis* may also facilitate research, toward fundamental biological processes of agricultural importance, in other non-climacteric fleshy fruits crops, such as melon or strawberry.

One of the greatest drivers for crop improvement is the resistance to stresses to which different genes are known to respond. For example, gene expression in both terpenoid and phenylpropanoid pathways in grapes is modulated under prolonged drought conditions (Savoi et al., 2016, 2017). A link to secondary metabolism is also seen by heat (Lecourieux et al., 2017) and UV-B/UV-C light stresses (Vannozzi et al., 2012; Carbonell-Bejerano et al., 2014). Stilbenoid-producing enzymes have been expressed in other plant species to confer resistance to fungal pathogens (Huang et al., 2018), which opens the possibility of biotechnological crop improvement in other species by using grapevine genes. All of the aforementioned stresses are a threat to crop yields and hence studying related gene families should be a key aspect of plant breeding strategies. A second motive for genetic improvement is fruit quality, which in turn affects wine characteristics of consumer interest such as flavor and aroma. For instance, anthocyanin accumulation in berry skins determines wine colour (Costantini et al., 2015). Genes providing these characteristics are often involved in the same metabolic pathways that respond to stresses.

**FIGURE 2:**
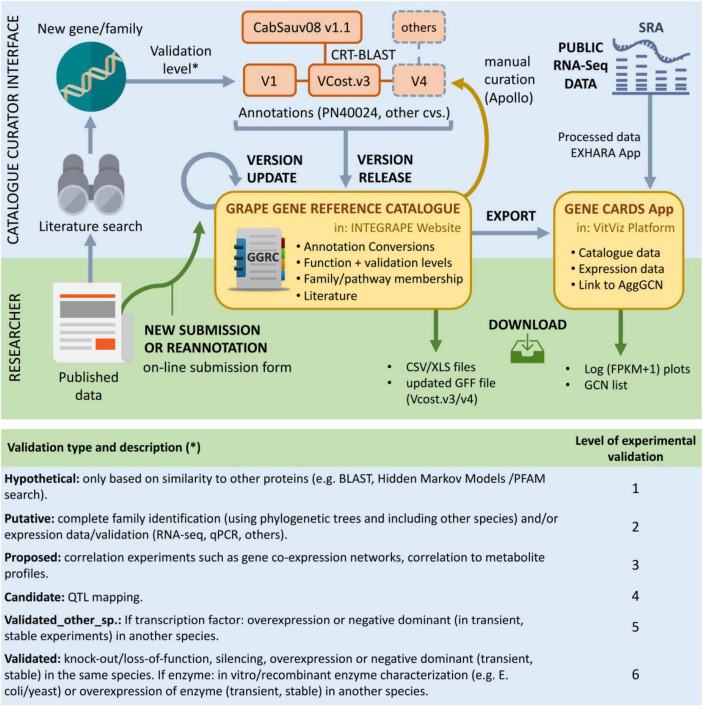
The grape gene reference catalogue in the context of published research, transcriptomic datasets, and genome annotation. The researcher interface allows to submit and download data (green arrows). Here, the catalogue will be updated with the online submission of new or corrected gene families. The updated catalogue will help in the manual curation of specific gene families using Apollo. It is also meant to reflect annotation changes from updated annotation versions resulting from manual curation. Gene correspondences with the available CabSauv08 v1.1 assembly are currently provided, whilst hoping to provide correspondences to other cultivars in the future. The catalogue information will also support the different tools provided by the VitViz platform such as Gene Cards or the Conversions app. Other tools include the visualisation of gene co-expression networks or the tissue-specific expression heat maps. Gene symbols are included in the latest 12X.2 assembly V.Cost gff annotation file and will also be present in the latest PN40024 40X.v4 assembly annotation. The lower panel shows the different levels of functional validation implemented in the catalogue.

A crucial aspect regarding the resources presented here is the participation of the grapevine community in both using and improving them (Figure 2). The development of the catalogue is designed to be a dynamic process through online submission of new or updated genes and gene families. Another key objective is to keep up with genome annotation updates and to assist community-wide manual curation of further gene annotation versions using browser-based tools for editing of sequence annotations such as Apollo (Lee et al., 2013). The VitViz platform will be periodically updated with this information, where different visualisation and analysis tools will benefit from the expanding knowledge.

With the objective of providing a high quality and highly accessible annotation of grapevine genes in the catalogue, we encourage the community to keep using the guidelines of the international Super-Nomenclature Committee for Grape Gene Annotation (sNCGGA) and their developed standard nomenclature for locus identifiers and conventions for a gene naming system (Grimplet et al., 2014). These guidelines (i) provide a common annotation platform that enables community-based gene curation, and (ii) develops a gene nomenclature scheme reflecting the biological features of gene products that is consistent with that used in other organisms in order to facilitate comparative analyses. Using this scheme is especially important when dealing with gene families, as one of the requirements to include them in the catalogue is the presence of robust phylogenetic trees in the referenced literature (i.e., appropriate use of bootstraps and inference methods).

A growing number of online resources are supporting crop communities. For instance, Gramene offers a cross-species view of genomics and metabolism in key species (Tello-Ruiz et al., 2021). Since Gramene also includes *Vitis*, improving grapevine resources will also have a positive impact on other crop platforms. We strive for a widespread use of these resources by the crop community and to this end we have engaged in Integrape COST Action CA17111 activities such as Working Group meetings.

## Data Availability Statement

Publicly available datasets were analyzed in this study. This data can be found here: INTEGRAPE (https://integrape.eu/) and VitViz (https://tomsbiolab.com/vitviz).

## Author Contributions

DN-P and JTM conceived the catalogue and wrote the manuscript. DN-P, AS, and LO contributed with the apps found in the VitViz platform and its connectivity with the catalogue through the Gene Cards app. CZ, AA, ED’I, CF, MP, GT, and SZ participated in the second release of the catalogue as part of the activities of a working group meeting of the Integrape Cost Action. All authors contributed to the article and approved the submitted version.

## Conflict of Interest

The authors declare that the research was conducted in the absence of any commercial or financial relationships that could be construed as a potential conflict of interest.

## Publisher’s Note

All claims expressed in this article are solely those of the authors and do not necessarily represent those of their affiliated organizations, or those of the publisher, the editors and the reviewers. Any product that may be evaluated in this article, or claim that may be made by its manufacturer, is not guaranteed or endorsed by the publisher.

## Footnotes

1
https://integrape.eu/resources/genes-genomes/

2
https://tomsbiolab.com/vitviz
